# Valorisation of Tannery Waste to Recover Chromium with a View to Reusing It in Industrial Practise

**DOI:** 10.3390/membranes14060136

**Published:** 2024-06-06

**Authors:** Anna Kowalik-Klimczak, Monika Łożyńska, Maciej Życki, Christian Schadewell, Thomas Fiehn, Bogusław Woźniak, Monika Flisek

**Affiliations:** 1 Łukasiewicz Research Network–Institute for Sustainable Technology, Pułaskiego St. 6/10, 26-600 Radom, Poland; monika.lozynska@itee.lukasiewicz.gov.pl (M.Ł.); maciej.zycki@itee.lukasiewicz.gov.pl (M.Ż.); boguslaw.wozniak@itee.lukasiewicz.gov.pl (B.W.); 2 Prüf- und Forschungsinstitut Pirmasens e.V., Marie-Curie-Str. 19, 66953 Pirmasens, Germany; christian.schadewell@pfi-biotechnology.de (C.S.); thomas.fiehn@pfi-biotechnology.de (T.F.); 3 Countrywide Chamber of Leather Industry, Włodzimierza Krukowskiego St. 1, 26-600 Radom, Poland; biuro@oibs.pl

**Keywords:** chromium waste, thermal pressure hydrolysis (TPH), ultrafiltration (UF), circular economy, tanning industry

## Abstract

This paper presents the basic assumptions of the concept of a new technology for the valorisation of chromium tannery waste. It assumes the use of an integrated system of the thermal pressure hydrolysis process and membrane filtration techniques for the recovery of chromium compounds and the use of a separated organic matter during anaerobic fermentation. According to the assumptions of the developed technological concept, at the first stage, the crushed mixture of chromium tannery waste is decomposed in the process of thermal pressure hydrolysis using appropriate process conditions in an alkaline environment. Then, the liquid product of this process (the so-called hydrolysate) is processed using centrifugal force separation and ultrafiltration. Such activities enable the recovery of chromium compounds for rawhide currying and concentration of organic matter (fats, proteins) with energy potential. Research carried out under conditions similar to real operating conditions proved that chromium compounds recovered from waste can be successfully used in the processing of cowhides intended for the production of footwear. The industrial implementation of the developed technology for valorising chromium tannery waste would enable the transition from a linear to a circular economy.

## 1. Introduction

Tanning is an essential process in leather processing because it prevents microbiological degradation. It employs mineral tannins, vegetable tannins, syntans, aldehydes, and fatty tannins, whose selection depends on the required properties of the finished leather, the cost of materials, and the type of raw material. The vast majority of the leather produced today (80–90%) is tanned using chromium salts [1,2,3].

According to statistical data, in 2020, approximately 10,000 tons of treated cowhides were produced in Poland, with a simultaneous production of 49.9 thousand tons of waste from the production of leather and leather products. Approximately only 2% of the waste was neutralized by the producer, and the remaining waste was transferred to other recipients. Additionally, 34 thousand tons of waste are stored on tannery premises [4,5]. Due to significant differences in the composition of waste generated in the tanning process, it is managed in different ways. Waste generated before the actual tanning process contains large amounts of proteins and fats. For this reason, it is relatively easy to manage the production of, among others, glues, feeds, soaps, and gelatine [6,7]. On the other hand, the management of chromium waste generated after tanning process is actually serious problem for tanneries. Therefore, numerous research and development units, both domestic and foreign, are conducting intensive work on developing an effective method for managing this type of waste. One of the technologies used for the management of chromium tannery waste is based on the use of an interphase transfer catalyst. It enables the recovery of chromium(III) in the form of inorganic compounds, which can be directly used during chromium tanning of raw hides. By-products of these processes, in the form of residues such as collagen, can be used to produce technical gelatine, organic fertilisers, or biopolymers [8]. However, this method involves high investment costs and, therefore, has not been commercialised yet. An alternative approach to managing chromium tannery waste is its thermal neutralisation, which enables energy recovery while reducing its volume. However, the disadvantage of this method is the release of carcinogenic chromium compounds. Only the use of a tunnel furnace and appropriately selected process conditions makes it possible to reduce the emission of dangerous waste gases and recover chromium, which can potentially be reused [9,10]. Interesting technological solutions are also the thermochemical method, which enables the use of chromium tannery waste to produce sorption materials intended for removing typical contaminants from wastewater [11] and ultrasonic extraction of chromium from tannery waste [12]. Recently, a method of disc granulation of tannery waste has been developed, the implementation of which in industrial practice could contribute to reducing the costs of waste storage, disposal, and transport. Its effectiveness has been confirmed for waste in the form of shavings [13,14]. Currently, chromium-based tannery waste is a significant challenge for leather producers who declare that they use the services of companies specialising in hazardous waste disposal. Unfortunately, this does not solve the problem because unmanaged chromium tannery waste remains in landfills, which is associated with the risk of carcinogenic forms of chromium(VI) entering the environment. For this reason, it is necessary to undertake further initiatives in material recycling to develop an effective method for managing chromium tannery waste [15].

The aim of this work was to develop a new concept of the valorisation of chromium tannery waste, which, in accordance with the circular economy model, will enable the recovery of valuable raw materials for reuse in the industry. This paper presents the research results for separating the chromium and proteins in the liquid phase resulting from the hydrolysis of chromium tannery waste. The impact of membrane cut-off on the efficiency of protein removal has been analysed.

## 2. Materials and Methods

### 2.1. Chromium Tannery Waste Treatment System

In the first stage, chromium tannery waste was subjected to thermal pressure hydrolysis (TPH). This process was carried out in a pressure reactor with a volume of 100 dm^3^ by Eugen Schmitt GmbH (Weselberg, Germany). The reactor feed was a mixture of chromium tannery waste (shavings, cuttings, and grinding dust) with a 1% sodium hydroxide solution. In the first pressure cycle of hydrolysis, the process was carried out at a temperature of 117 °C, while the next/final stage was carried out at a temperature of 160 °C for 60 min. After this time, the reactor was left to cool down. TPH was carried out using an input consisting of a mixture of 2 kg of waste and 10 kg of sodium hydroxide solution. A total of 5 to 10 kg of steam was used for this process (taking into account the heating and cooling processes). As a result of TPH, a liquid was created (‘alkaline hydrolysate’). It was subjected to centrifugal separation. The process was carried out on an AFI Loreena centrifuge for 20 min at 2000 rpm. Then, the overlying liquid was subjected to the ultrafiltration (UF) process used for laboratory membrane setup. This filtration system consists of a cross-flow membrane cell, feed/permeate/retentate tanks, thermostat, pressure pump, manometer, and flowmeter. The schema of laboratory installation was presented in the work in [16]. In turn, the general specifications of the cross-flow membrane cell are presented in Table 1.

The liquid was introduced into the feed tank, from where the pump forced it into the cross-flow membrane cell, where it was separated into a permeate collected in a separate tank, and the retentate returned to the feed tank. For each UF process, 4 dm^3^ of the feed was used. The UF process continued until the permeated amount of 2.4 dm^3^ was received. This means that the VRF for each UF process was equal to 2.5. During the process, the temperature in the feed/retentate container was kept at 25 ± 1 °C. UF processes were carried out with polyethersulfone (PES) membranes by Synder—MT and MQ MAX, which differ in the separation limit (cut-off). The smallest molecular weight of the substance retained by the MT membrane at a level no lower than 90% was 5000 Da. In turn, in the case of the MQ, the MAX membrane was ~50,000 Da. Both types of membranes used were characterised by high chemical resistance (pH in the range of 1–11 for the MT membrane and pH in the range of 1–13 for the MQ MAX membrane) and resistance to operation at a maximum temperature of 45 °C. UF membranes were selected based on the data provided by their manufacturer. This type of membrane is commonly used to separate proteins from industrial liquids. The purpose of their use was to separate proteins present in the liquid phase resulting from the hydrolysis of chromium tannery waste. The impact of the membranes’ cut-off on the efficiency of protein removal from alkaline hydrolysates is analysed in this work.

### 2.2. Physical and Chemical Parameters of Waste, Hydrolysates, and Treatment Products

To assess the degree of retention of individual components in the integrated thermal hydrolysis/centrifugal force separation/membrane filtration system, all resulting streams were subjected to multi-parameter physicochemical analysis. The pH and conductivity of the solutions were examined using a multi-channel Seven Excellence meter by Mettler Toledo enabling a simultaneous analysis of both parameters. The analysis of total chromium concentrations performed using inductively coupled plasma emission spectrometry ICP-OES by a Perkin Elmer Optima 5300 V (LOD_Crtotal_ equal 5.0 µg/dm^3^) was preceded by sample mineralisation. Pressure mineralisation was carried out in a closed system supported by microwave radiation in a mixture of H_2_SO_4_ and H_2_O_2_. However, to determine the concentration of chromium(VI), the samples were leached with a NaOH/Na_2_CO_3_ solution and then determined using the spectrophotometric method with 1,5-diphenylcarbazide. Chemical oxygen demand was determined by high-temperature combustion using a QuickCODlab analyser by LAR. The content of total nitrogen bound and total organic carbon was determined using a Vario TOC cube analyser by Elementar, with a catalytic combustion method in an oxygen stream. The concentration of chlorides and sulphates was determined using spectrophotometric methods with dedicated cuvette tests and a UV-VIS DR 6000 spectrophotometer by Hach Lange (Loveland, CO, USA). Dry matter and organic dry matter contents were determined using gravimetric methods by drying at 105 °C and roasting at 550 °C to constant mass, respectively. An FD 23 laboratory dryer by Binder (Tuttlingen, Germany), a FB1410M-33 muffle furnace by Thermo Scientific, and an EX324M analytical balance by Ohaus (Nänikon, Switzerland) were used for these tests.

### 2.3. Model Tanning Tests

Bovine hides were tanned after deliming. The tanning process was carried out using a traditional industrial method employing a pickling bath. Model leather tanning tests were carried out on an industrial scale. In the leather tanning processes, a mixture of commercial Chromitan B tanning agent (5%) and chromium recovered from tannery waste (3%) was used. The reference sample was leather-tanned with a commercial Chromitan B tanning agent (7%). Once the tanning process was completed, the leather samples were removed from the drums and left for 48 h, after which their physicochemical parameters were tested (thickness, tensile strength, elongation, tensile force, bulge height, finish adhesion, chromium(III) content, humidity). The standards and methods used to determine them were described in a previous work [17].

### 2.4. Microscopic Tests

The microscopic examinations carried out included observations of chromium deposits isolated from the hydrolysate, polymer membranes, and bovine hides. To image their surface, a Hitachi SEM field emission scanning electron microscope with a Schottky thermal emitter, model SU-70 (Hitachi, Tokyo, Japan), was used. In parallel, microscopic observations of the surface of bovine leather samples were carried out using a Keyence VHX-6000 optical microscope (Keyence, Mechelen, Belgium).

## 3. Results and Discussion

### 3.1. Hydrolysis of Tannery Waste Containing Chromium

The research concerned chromium tannery waste (in the form of cuttings, shavings, and dust), characterised by physicochemical parameters presented in the previous paper [17]. In the first stage of this work, chromium tannery waste was subjected to thermal pressure hydrolysis (TPH) carried out in an alkaline solution. The product of the TPH of chromium tannery waste carried out in a sodium hydroxide environment, i.e., the so-called alkaline hydrolysate, was characterised by the physicochemical parameters listed in Table 2.

### 3.2. Hydrolysate Treatment Using Separation Processes

**Figure 1:**
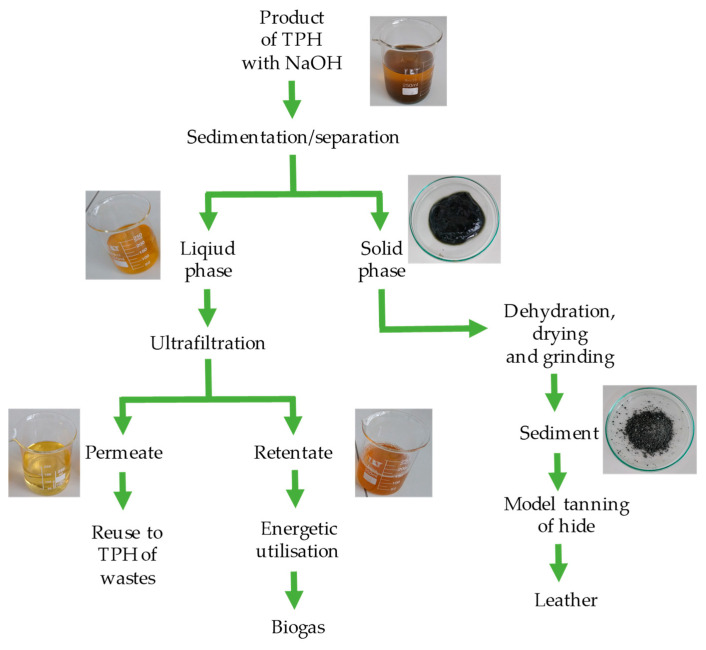
Fractionation procedure for a liquid fraction created as a result of TPH of chromium waste from tanneries carried out in a basic environment.

The alkaline hydrolysate was processed according to the procedure presented in Figure 1.

**Figure 2:**
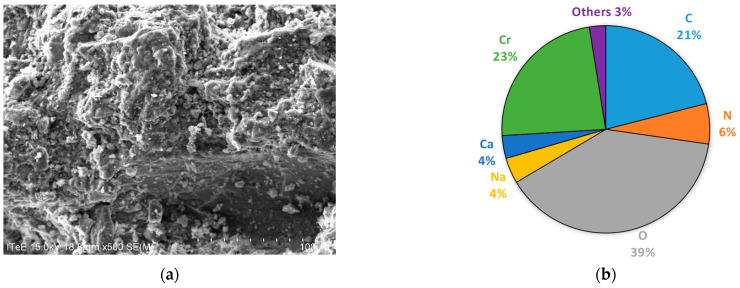
Images of SEM (**a**) (magn. ×500) and elemental composition of EDS analysis (**b**) of the solid phase isolated from hydrolysate.

According to the procedure presented in Figure 1, a precipitate was separated from the alkaline hydrolysate, which, after drying and grinding, was imaged using a scanning electron microscope (Figure 2a) and examined for its composition using the EDS technique (Figure 2b). Based on the analysis of the test results, it was found that the sediment mainly consists of chromium, oxygen and carbon.

**Figure 3:**
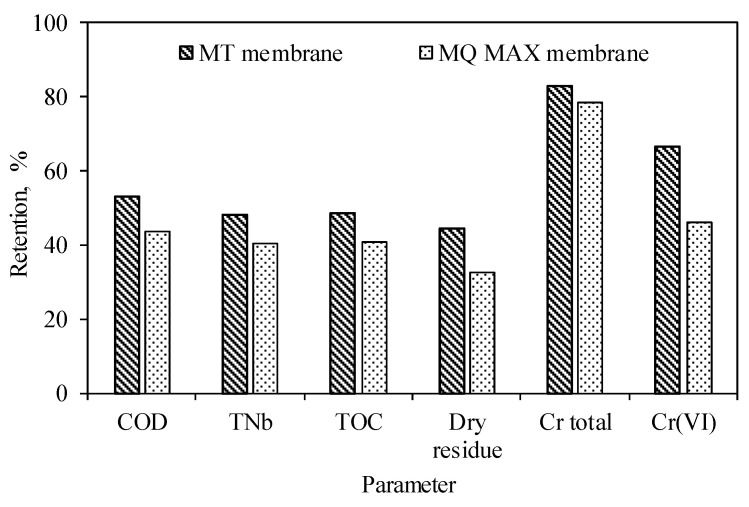
Retention of individual components of alkaline hydrolysates during ultrafiltration carried out using two types of membranes.

The overlying liquid (Figure 1) was subjected to membrane separation. The ultrafiltration process conducted at a pressure of 4.0 bar and a feed flow of 180 dm^3^/h was characterized by an efficiency of 77.3 ± 7.1 dm^3^/(m^2^h) and 66.6 ± 7.7 dm^3^/(m^2^h) for the MT and MQ MAX membranes, respectively. During these processes, a decrease in efficiency of 26% (MT membrane) and 18% (MQ MAX membrane) was observed. This is because the structure of the MT membrane is more compact than that of the MQ MAX membrane. This is evidenced by higher retention factors of components characteristic of alkaline hydrolysates recorded for the MT membrane (Figure 3). The use of the MT membrane (cut-off~5000 Da) enabled the higher retention of components responsible for organic matter (i.e., chemical oxygen demand—COD, total nitrogen bound—TNb, total organic carbon—TOC, dry residue) and total chromium and chromium(VI) than in the case of the MQ MAX membrane (cut-off~50,000 Da).

Moreover, both membranes did not retain mono- and multivalent ions (chlorides and sulphates). They allowed for a small reduction in the dry organic matter content (by approx. 14% for the MT membrane and approx. 10% for the MQ MAX membrane). As a result of using a system of centrifugal separation and ultrafiltration processes, concentrates of organic matter were obtained (COD concentration of 87.9 ± 2.5 g O_2_/dm^3^ and 81.7 ± 0.9 g O_2_/dm^3^ for the MT and MQ MAX membranes, respectively) characterised by a low content of total chromium (concentrations of total chromium of 21.5 ± 0.3 mg/dm^3^ and 20.4 ± 0.3 mg/dm^3^ for the MT and MQ MAX membranes, respectively). The aim of using the UF process was to separate organic compounds from the alkaline hydrolysate. This process was selected based on literature data [18,19], according to which it can be successfully used for the preliminary treatment of tannery wastewater. During the UF process of alkaline hydrolysates, partial separation of chromium was observed (Figure 3). According to research conducted by Muthumareeswaran et al. [20], it is possible to remove over 90% of chromium(VI) from alkaline solutions. Still, the separation ability of UF membranes depends on the initial concentration of chromium in the solution and the applied process parameters (cross-flow velocity and pressure). In turn, research conducted by Mert et al. [21] proved the possibility of the partial retention of total chromium during the UF of tannery wastewater.

The hydrolysate subjected to the UF process contained 13.70 ± 0.42 mgCr_total_/dm^3^. After the UF process, the retentate amounted to 21.50 ± 0.28 mgCr_total_/dm^3^ and 20.40 ± 0.28 mgCr_total_/dm^3^ for the MT and MQ MAX membranes, respectively. Based on the results of research conducted by Ding et al. [22], a negative impact of the increase in the concentration of chromium present in the raw material undergoing anaerobic fermentation on the efficiency of biogas production was found. The concentration of chromium in the biogas raw material at the level of 20 mg/dm^3^ causes the maximum daily gas production to be reduced by approximately 58% compared to the raw material not containing this element. Anaerobic fermentation of raw materials containing chromium leads to the production of biogas with a composition similar to biogas produced on the basis of chromium-free raw materials. Still, it may result in the formation of volatile fatty acids. Similar results of research on the anaerobic fermentation of chrome and chrome-free tanning waste were obtained by Fernández-Rodríguez et al. [23]. Therefore, the possibility of using the retentate from UF carried out on the hydrolysate from the TPH process of chromium tannery waste is considered to be in the anaerobic co-digestion of chrome-free tannery waste, the high biogas potential of which was described in the works of Agustini et al. [24] and Mia et al. [25].

### 3.3. Membrane Fouling

**Figure 4:**
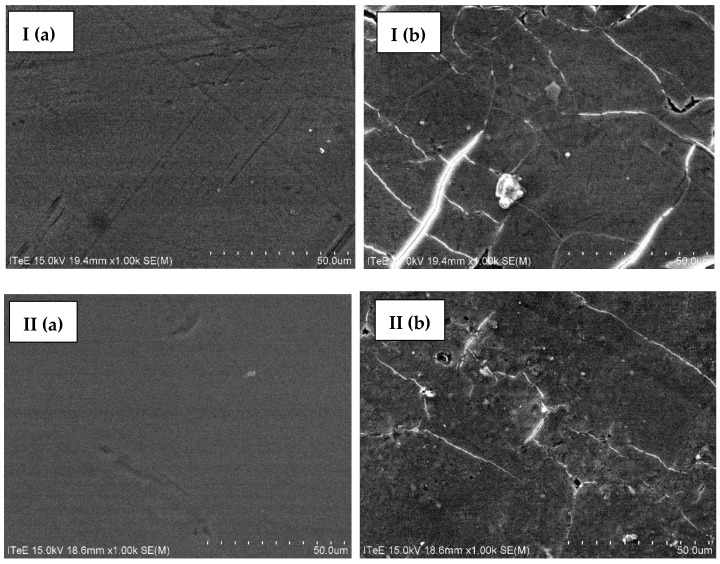
Images of the surface of (**I**) MT and (**II**) MQ MAX membranes before (**a**) and after (**b**) ultrafiltration of liquid fraction created as a result of thermal pressure hydrolysis of chromium waste from tanneries, pre-treated using centrifuge, taken with an SEM microscope (magnification: ×1000).

**Figure 5:**
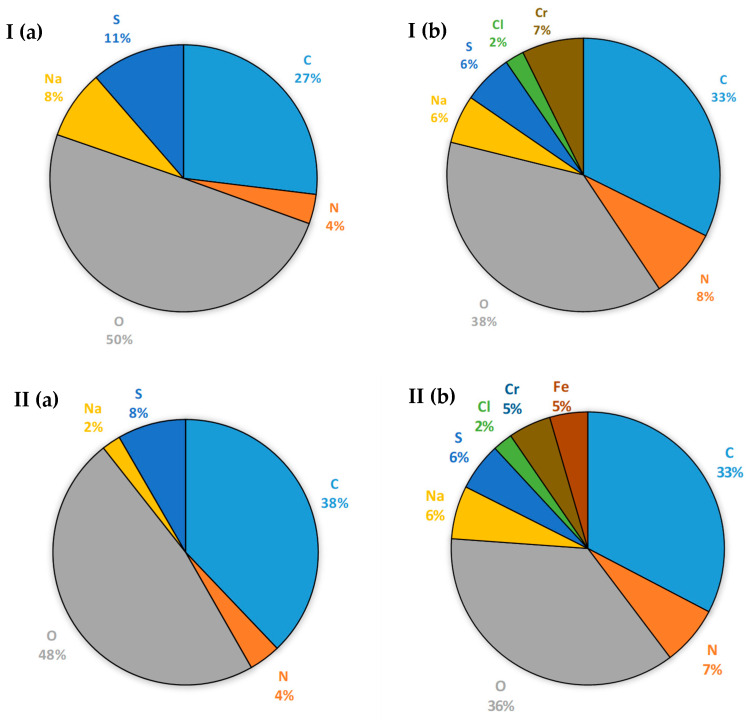
Elemental composition (%) of the surface of (**I**) MT and (**II**) MQ MAX membranes before (**a**) and after (**b**) ultrafiltration of liquid fraction created as a result of thermal pressure hydrolysis of chromium waste from tanneries, pre-treated using centrifuge, determined using EDS.

Ultrafiltration membranes were tested for changes in surface structure. For this purpose, both types of membranes before and after the ultrafiltration process were imaged using SEM (Figure 4), and the composition of their surface was determined using the EDS technique (Figure 5).

Based on the research results, it was found that the surface structure of MT and MQ MAX membranes changes under the influence of the settling components of the filtered liquid (Figure 4). The sediment deposited on the surface of the MT membrane consists mainly of chromium and chlorine (Figure 5 I(b)). In turn, the sediment that was adsorbed on the surface of the MQ MAX membrane consists mainly of chromium, chlorine, and iron (Figure 5 II(b)). Taking into account the efficiency of the process and the rate of decline in the filtration stream during it, it was found that it is beneficial to use an MQ MAX membrane for ultrafiltration of the liquid generated during the hydrolysis of chromium tannery waste in an alkaline environment.

### 3.4. Model Bovine Hide Tanning Test Using Chromium Recovered from Tannery Waste

**Figure 6:**
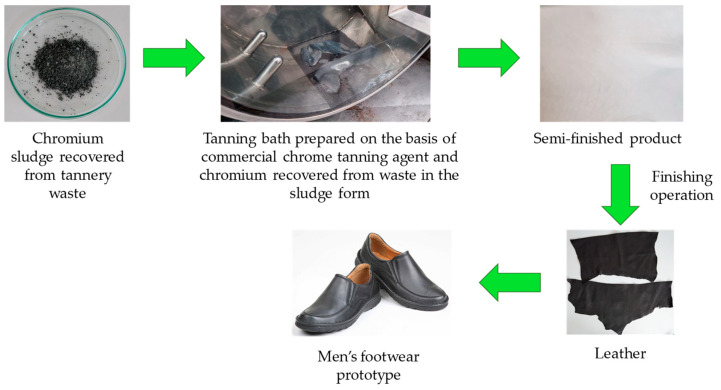
Procedure for using chromium recovered from waste generated during tanning of hide.

The procedure for using chromium recovered from waste during the tanning of raw hides is shown in Figure 6. The sludge retrieved from the waste was dissolved in sulfuric acid(VI) and used to prepare the tanning bath. Model leather tanning tests were carried out using a mixture of this liquid (3%) and a commercial chromium tanning agent (5%). The reference sample was cowhide-tanned in a traditional way using commercial chromium tannin (7%). During the visual assessment of the samples, it was found that the semi-finished product treated with the use of liquid, which was created by dissolving the precipitate of chromium compounds after alkaline hydrolysis in sulfuric acid(VI), is grey-blue in colour, which is characteristic of traditionally tanned leather. Thanks to this, it can be dyed in any colour. In the next stage of the research, both leather semi-finished products were subjected to finishing operations. The finished leather was used to create a prototype of men’s shoes for everyday use.

The physicochemical parameters of leather tanned using chromium recovered from waste are listed in Table 3. The test results were compared with the physicochemical parameters of leather tanned traditionally using a commercial chromium tanning agent. Based on the results of physical and chemical tests, it was found that the physical and chemical characteristics of leather tanned with the addition of chromium recovered from waste are similar to those of traditionally tanned leather. Hence, it can be claimed that the economic value of leather is not affected when the leather is tanned with the addition of 3% recovered chromium to the tanning bath. Similar results were obtained by Khan et al. [26] during tanned hides by chromium recovered from spent tanning baths by the precipitation process. Drioli et al. [18] proposed the regeneration of the chromium spent tanning bath with an integrated UF/NF system. The waste chrome liquor treatment method using solar evaporation also shows promise as a practical solution for valorisation [27]. The results of research and literature data [18,26,27] imply that the recovered chrome can be seamlessly incorporated into subsequent tanning processes without compromising the economic value of the resulting leather. Considering this, it is expected that the tanneries will be interested in a circular economy setup chrome recovery plant to ensure a protected environment and a profitable chrome tanning process.

**Figure 7:**
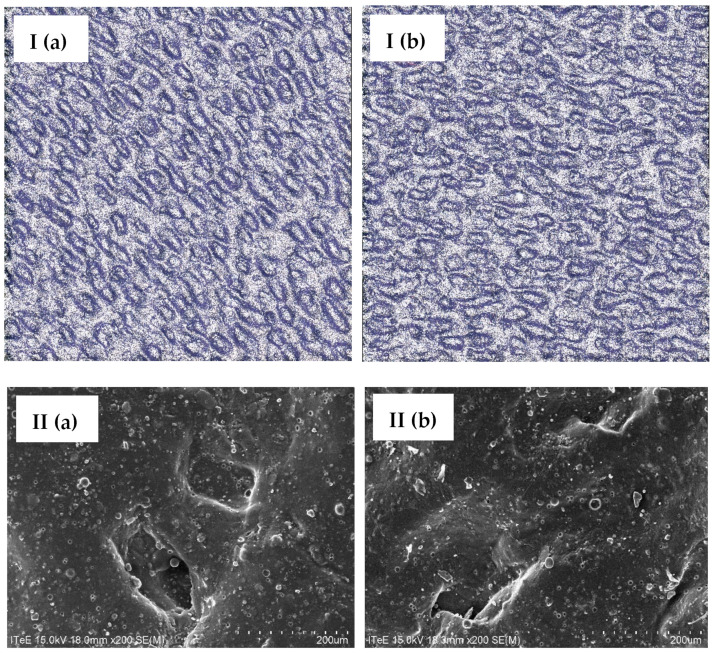
Images of the surface of bovine hides tanned using (**a**) a commercial tanning agent and (**b**) chromium recovered from tannery waste (sediment), taken using the (**I**) 3D microscope (magnification: ×100) and (**II**) SEM (magnification: ×200).

**Figure 8:**
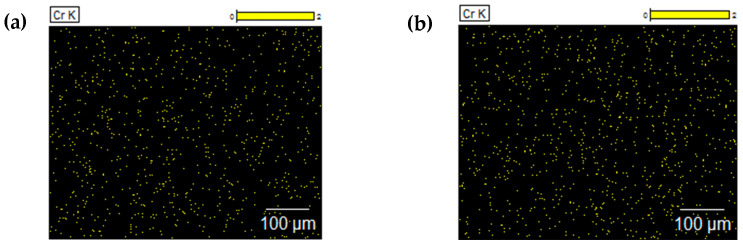
Maps of chromium distribution on the surface of bovine hides tanned using (**a**) a commercial tanning agent and (**b**) chromium recovered from tannery waste (sediment), taken using EDS.

Leather samples tanned using chromium recovered from waste were subjected to microscopic observations and compared with microscopic images of leather tanned traditionally using a commercially available chromium tanning agent. The research used a 3D microscope and a scanning electron microscope (SEM). Images of leather tanned traditionally and with the addition of chromium recovered from waste and a map of the distribution of chromium on the surface of the leather are presented in Figure 7 and Figure 8. Based on the microscopic observations, it was found that the use of chromium recovered from waste does not have a negative impact on the structure of the leather surface. Moreover, the distribution of chromium on the surface of leather tanned using chromium recovered from waste was even and similar to the distribution of chromium on the surface of traditionally tanned leather.

The analysed physicochemical parameters of leather tanned traditionally and with chromium recovered from waste enable its use in the production of durable everyday footwear for women and men (Figure 6). At the next stage of the research work, the hygienic and functional properties of the manufactured footwear, such as water permeability, resistance to weather conditions, or health safety, will be tested [28,29]. Positive results of this type of tests would enable obtaining the approvals necessary to implement the technological solution developed in this work for industrial production.

## 4. Conclusions

The paper presents a technological concept for valorising chromium tannery waste, which uses an integrated system of thermal pressure hydrolysis and separation processes. In the first stage, the crushed mixture of unusable scraps of leather after chromium tanning is decomposed in the process of thermal pressure hydrolysis using appropriate process conditions in a sodium hydroxide environment. Then, the product of this process (so-called alkaline hydrolysate) is fractionated using centrifugal force separation and ultrafiltration. The centrifugal force separation process enables the separation of chromium deposits for reuse in industrial practice during the tanning of raw hides. In turn, the ultrafiltration process enables the recovery of a concentrate of organic matter with energy potential. The obtained research results are promising and create the prospect of implementing the principles of clean production in enterprises of the tanning industry.

## Figures and Tables

**Table 1:** The general specifications of the cross-flow membrane cell.

Parameter	Value
Outer Dimensions	16.51 × 21.3 × 5 cm
Active Area Dimensions	9.7 × 14.7 cm
Slot Depth	0.19 cm
Slot Width	95.3 mm
Material	316 Stainless Steel

**Table 2:** Physical and chemical parameters of the liquid fraction created as a result of the thermal pressure hydrolysis (TPH) of chromium waste from tanneries carried out in a basic environment.

Parameter	Value
pH	9.433 ± 0.029
Conductivity (mS/cm)	19.451 ± 0.012
Total chromium (mg/dm^3^)	552.0 ± 2.8
Chromium (VI) (mg/dm^3^)	2.801 ± 0.084
Chemical oxygen demand (COD) (g O_2_/dm^3^)	69.52 ± 0.69
Total organic carbon (TOC) (g/dm^3^)	19.24 ± 0.33
Total nitrogen bound (TNb) (g/dm^3^)	10.68 ± 0.10
Chlorides (g/dm^3^)	2.697 ± 0.092
Sulphates (g/dm^3^)	4.212 ± 0.094
Dry residue (%)	6.320 ± 0.026
Organic dry residue (% dry matter)	73.34 ± 0.24

**Table 3:** Comparison of the physical and chemical parameters of hides tanned in a traditional manner and using chromium recovered from waste subject to basic hydrolysis.

Parameter	Sample 1	Sample 2
Thickness (mm)	1.27	1.27
Tensile strength (N/mm^2^)	22.64	21.86
Elongation (%)	50	47
Tear strength (N)	92.83	91.38
Brusting factor (lastometer)	9.9	9.5
Adhesion of finish (N/cm]	4.2	5.4
Chromium(III) content converted to Cr_2_O_3_ (%)	4.33	4.18
Sample 1	Hide tanned using a commercial tanning agent	
Sample 2	Hide tanned using chromium recovered from waste subject to basic hydrolysis	

## Data Availability

The original contributions presented in the study are included in the article, further inquiries can be directed to the corresponding author.
